# Chemistrees: Data-Driven Identification of Reaction Pathways *via* Machine Learning

**DOI:** 10.1021/acs.jctc.1c00458

**Published:** 2021-09-24

**Authors:** Sander Roet, Christopher D. Daub, Enrico Riccardi

**Affiliations:** † Department of Chemistry, Norwegian University of Science and Technology, Høgskoleringen 5, 7491 Trondheim, Norway; ‡ Department of Chemistry, University of Helsinki, P.O. Box 55, FI-00014 Helsinki, Finland; § Department of Informatics, UiO, Gaustadalléen 23B, 0373 Oslo, Norway

## Abstract

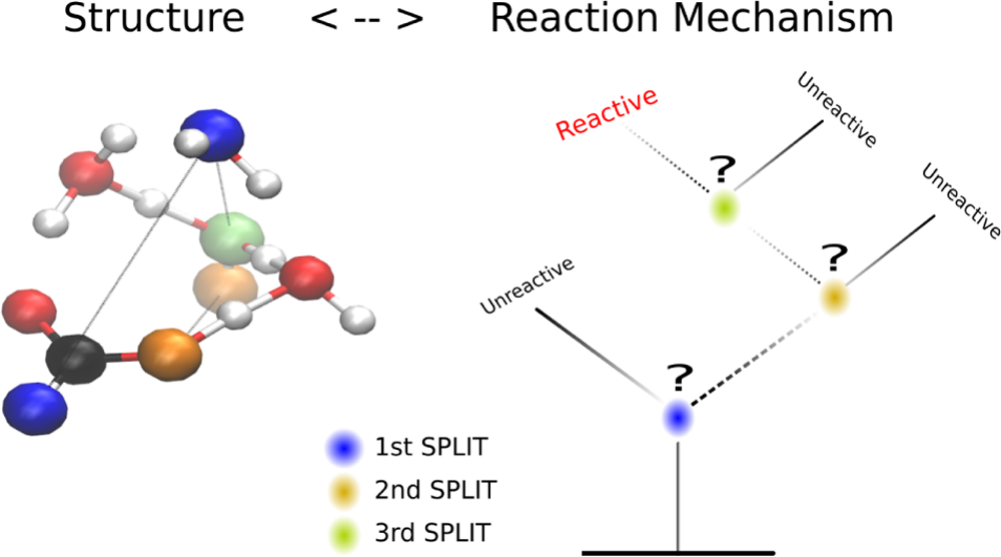

We propose to analyze molecular dynamics (MD) output *via* a supervised machine learning (ML) algorithm, the decision tree. The approach aims to identify the predominant geometric features which correlate with trajectories that transition between two arbitrarily defined states. The data-driven algorithm aims to identify these features without the bias of human “chemical intuition”. We demonstrate the method by analyzing the proton exchange reactions in formic acid solvated in small water clusters. The simulations were performed with *ab initio* MD combined with a method to efficiently sample the rare event, path sampling. Our ML analysis identified relevant geometric variables involved in the proton transfer reaction and how they may change as the number of solvating water molecules changes.

## 1 Introduction

In regions far from urban areas, formic acid (FA) has been recognized as one of the main factors which reduces the pH of rainwater, causing acid rain.^1^ It has relatively high atmospheric concentrations^2,3^ and contributes to the formation of sulfuric acid in the atmosphere.^4,5^ Enhanced description of proton exchange reactions involving solvated FA can improve the current atmospheric models. Theoretical studies of proton transport in bulk aqueous media have a long history going back to the elucidation of the Grotthuss mechanism.^6^ The current view of the solvated proton in water focuses on the formation of Zundel (H_5_O_2_^+^) and Eigen (H_9_O_4_^+^) cations and the mechanisms describing transformations between these states.^7−13^

A related area with significant theoretical and computational contributions in the last decade is the study of acid ionization in bulk water^14−18^ or at the water–air interface.^9,19−23^ By contrast, there are only a few papers which focus on the nature of acidic proton transport in small water clusters.^24−30^ In these small systems, thermodynamic approaches appropriate for the bulk system are no longer valid. Instead, these studies have been forced to approach each specific chemical example as a separate problem. As such, the use of a generalizable approach such as the one we present in this study should be of considerable interest.

*Ab initio* molecular dynamics (MD) simulations have recently been used to examine FA deprotonation in aqueous solution,^18,22^ successfully describing the proton exchange reaction between water and FA. While these studies led to valuable new insights, the limitations of the adopted methods (*e.g.*, usage of a bias potential and continuous collective variables) could be overcome, thanks to relatively novel methodologies such as replica exchange transition interface sampling (RETIS).^31,32^ Respecting the natural dynamics of the system, it allows the study of transitions even with a significant diffusive contribution^33,34^ (*i.e.*, a small reaction barrier) and enables the direct investigation of reaction mechanisms.

RETIS is a rare event method developed to investigate transitions. Its main advantages are as follows: (a) it does not alter the natural dynamics of the system, (b) it does not require a particularly accurate order parameter, (c) its results are in principle identical to what would be obtained by an infinitely long unbiased MD simulation. With RETIS, the transition region is explored by continuously generating new paths which start from a stable state and end up either back in such a state (an *unreactive* path), or reach a different state (a *reactive* path). The approach has been successfully employed to study transitions that would, otherwise, require prohibitively long simulation times. The results generated have been used to describe the dynamics of chemical processes (*e.g.*, reaction rates) while considering the entropic contribution in the analysis.^5,33−36^ Since significant amounts of data are often generated by the sampling procedure, approaches to pragmatically decode reaction mechanisms are greatly beneficial.

Our aim is to establish a heuristic approach to describe transitions regardless of whether they involve crossing an entropic barrier. Data-driven, physically consistent, and measurable system descriptors might be generated and their correlations with the system dynamics asserted. It is a classification problem, which a machine learning (ML) algorithm can be trained to solve. The algorithm might then predict if a certain molecular structure (frame) is part of a reactive or a non-reactive trajectory. Connecting the descriptors to measurable quantities provides a data-driven “unbiased” description of a transition that might support, and eventually surpass, human-biased “chemical intuition”.

Data-driven algorithms for enhanced sampling or the analysis of chemical simulations have significant recent contributions.^37−43^ Most of these approaches are based on neural networks, which lack physically consistent interpretability, which is, instead, a characteristic of decision trees (DTs).^39,40^ Furthermore, in most of these studies implementing neural networks, a pre-selection of trial collective variables^38,41,42^ is required, which could lead to a hypothesis-bias. DT^44^ classifiers have a unique solution and are not sensitive to highly correlated variables. The results can be readily interpreted if the source variables are also interpretable. The approach was previously adopted to select optimal collective variables with DTs, with reasonable success.^36^

We here propose a method based on DTs, which is both interpretable and hypothesis-bias-free *via* an appropriate system representation invariant to system translation, rotation, and changes in atomic indices. Our aim is to gain insights into reaction mechanisms with a systematic and objective representation of the system.

The approach has been developed with sufficient versatility to be applied to different types of molecular simulations, from conventional MD to rare event methods. It should be noted that conventional MD would require *a priori* classification of the data, that is, dividing the source trajectory into reactive and unreactive segments. The sampling strategy of rare event methods, instead, generates a data structure which inherently classifies the trajectories. Regardless of the adopted molecular simulation approach, limiting the correlation between samples is a primary task for a quantitative data-driven method to identify reaction paths and the probability of their occurrence.

We demonstrate our data-driven method in this study on small clusters of FA solvated by water, HCOOH + (H_2_O)_*n*_, *n* = 4 and 6. The system is relatively small and well understood and hence provides an ideal test case for training an ML method. Our analysis provides new quantitative and qualitative insights into the acid–water proton transfer reaction in aqueous clusters.

## 2 Computational Models and Methods

Since the main focus of the present paper is an ML methodology, we provide only a brief introduction to the simulation methodology. Please consider our previous studies^5,18^ for further details.

### 2.1 System Description

For studying proton transport, molecular simulations able to consider bond formation and bond breaking are required. Born–Oppenheimer MD has been shown to be a suitable approximation in previous studies of atmospheric reactions^4,5^ and of aqueous FA.^18,22^ The density functional theory BLYP, implemented in the Quickstep module of CP2K,^45^ has been adopted with a double-zeta basis set supplemented by the use of Grimme’s D2 dispersion correction.^46^

**Figure 1:**
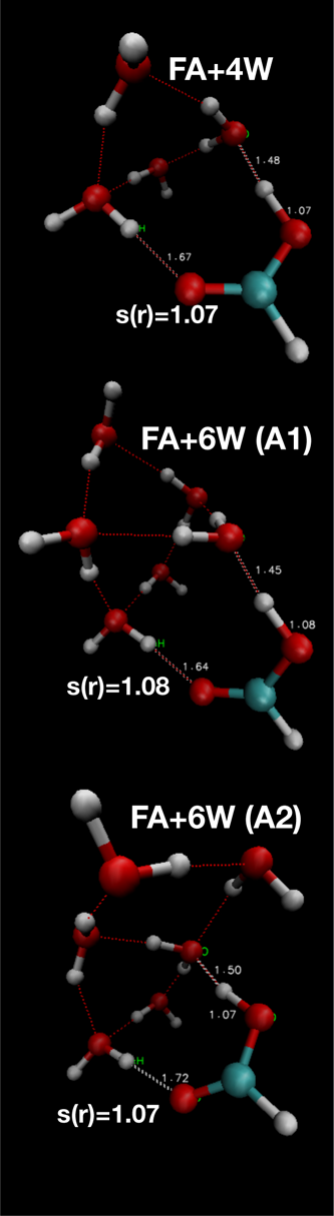
Minimum energy configurations for systems with FA associated with four and six water molecules. In the figures, *s*(*r*) is equal to the *r*_OH_ of the initially protonated FA molecule. These configurations are the initial states used to initiate PyRETIS simulations.

A set of systems with an increasing number of water molecules around FA were studied. Initial configurations were obtained from minimum energy configurations, of which snapshots are reported in Figure 1. As more water molecules were added, the probability of generating reactive trajectories increased. However, at least four added water molecules were required to allow generation of trajectories with a significant charge separation between the deprotonated FA and the solvated proton. With two additional water molecules, a significantly higher proton transfer rate was measured. The two systems composed by FA surrounded by four and six water molecules have been thus selected and discussed here.

### 2.2 Definition of the Collective Variable

Path sampling simulation requires the definition of a collective variable, *s*(*r*), to quantify the progress of a transition (*r* contains the positions and velocities of all atoms in the system). The method is not limited to continuous collective variables, allowing the consideration of relatively complex functions to describe proton transport.

The collective variable adopted in the present work is inspired by the study of water ionization,^36^ with modifications introduced to consider acid deprotonation. As a first step, it locates the smallest distance between any FA oxygen and any reactive hydrogen in the system (excluding the methyl hydrogen in FA). This distance is denoted as *r*_O_FA_H,min_.

For *r*_O_FA_H,min_ < 1.4 Å, FA is considered protonated, so *s*(*r*) = *r*_O_FA_H,min_. For *r*_O_FA_H,min_ > 1.4 Å, charge separation between the solvated proton and FA becomes significant. To quantify it and thus compute *s*(*r*), all the distances between reactive hydrogens and oxygens are first calculated. Hydrogens are then assigned to the closest oxygen, either water or FA. Any water oxygen found to be associated with three hydrogens is then indexed. All distances between FA oxygens and hydrogens associated with triply coordinated water oxygens are finally sorted. *s*(*r*) is the minimum value of these distances.

Conceptually, we aimed to describe the formation of complexes resembling Eigen or Zundel cations. A discontinuous jump of *s*(*r*) from ∼1.8 up to ∼3 Å is associated with a change in the identity of the triply coordinated water oxygen and the formation of structures resembling Zundel cations H_5_O_2_^+^. The formation of the Zundel cation with *s*(*r*) > 2.9 Å is here labeled as the product state B.

### 2.3 RETIS

The PyRETIS^47,48^ library has been used to perform RETIS^49^ simulations coupled with the *ab initio* MD external engine CP2K. In the four-water simulations, the first interface was placed at *s*(*r*) = 1.05 Å and the last interface at *s*(*r*) = 3.0 Å, thus defining the initial and the final states of the transition. Seventeen interfaces were positioned along the interval. Similarly, the six-water simulations had the first interface at *s*(*r*) = 1.07 Å and the last interface at *s*(*r*) = 3.0 Å. Thirteen interfaces were positioned along the interval.

The initial paths describing the transition from protonated to deprotonated FA along *s*(*r*) were generated by using the *kick* method available in the software, starting from the initial configurations shown in Figure 1. The “*kick*” approach uses a mixture of stochastic and deterministic dynamics to generate a set of initial paths. From the results, the paths that correlated with the initial generated ones were discarded. Finally, the remaining trajectories from a set of multiple independent simulations were merged together for both the four- and six-water-molecule cases.

### 2.4 Selection Window

In a trajectory, each frame can be considered as an instance in a data-representation suitable for the DT. Depending on the simulation setup, a large number of frames would generate a long list of instances with a very high correlation. Furthermore, different trajectories can be highly correlated with one another, depending on the sampling algorithm. Since generating a sufficient number of uncorrelated trajectories often requires excessive computational requirements, an approach to provide a sufficient sampling with a limited correlation is proposed here.

Frames contained in a rather restricted region in the path space can be identified *via* a selection window. By randomly picking a certain number of frames for each trajectory, within the selection window, the correlation between instances is minimized. By placing the selection window in proximity to the initial state, as in the current study, the system configurations which are correlated with the transitions can be identified prior to the transition actually occurring. The selection window location and dimension and the number of frames per trajectory to consider constitute the three hyper-parameters of our approach. In the present work, the ML algorithm has been fed with one frame per trajectory within a selection window defined by values of the order parameter 1.1 < *s*(*r*) < 1.25 Å. The range is sufficiently narrow to consider only a few frames for each trajectory, each with a similar order parameter. The ML algorithm should, therefore, be able to determine the most relevant feature(s) associated with the transition happening without hypothesis-bias on the main descriptor of the transition itself. This limits the correlation of the detected features with the classification of the trajectory.

### 2.5 Training the DTs, Labels

The ML problem we are posing is as follows: “what are the main features that a simulation frame has to have in order to be part of a trajectory that connects an initial state to a final state (reactive)?” and “with which probability?” The information gain (entropy) DT is a viable method for a problem with highly correlated features.^50^

DTs report the most important features that differentiate between reactive and unreactive paths without imposing any prior hypothesis.

Given a set of trajectories, a classification between reactive and unreactive paths is first needed. A numerical descriptor, conventionally defined as the order parameter, can quantify the progress of a given transition. If its value for a given system is within certain arbitrarily defined ranges, the system can be considered to be located in the initial or the final state. A reactive path is defined as a path starting from an initial state and ending at a product state. A non-reactive path, instead, ends at the initial state.

If the input generated by molecular simulation is composed of a single long trajectory, sub-segments will have to be fed to the ML task. In such a case, a segment starting at one state and ending in another state will be considered reactive, whereas a segment starting and ending at the same state without having previously entered another state will be unreactive. When using the input generated by path sampling, paths contained in a single ensemble should be considered (please consider refs (31) and (51) for the definition of an ensemble and further details of the path sampling methods).

### 2.6 Training the DTs, Data Matrix

Generally, all trajectory segments or trajectories for path sampling can be considered in the present analysis approach. When using path sampling, a re-weighting algorithm is adopted to consider all the generated paths. Due to the statistical weights of the different ensembles and for simplicity, we opted to consider only the trajectories included in the outermost ensemble in the path space (for the definition of an ensemble, please consider the RETIS formalism^49^).

From molecular simulations, an ordered data array for the positions and velocities for each atom is written for each selected time frame. While the convention facilitates post-processing and visualization procedures, it includes a bias in the data representation. Small deviations in the observation angle or on the choice of coordinate system (*e.g.*, exchanging *x* with *y* coordinates) lead to significantly different data sets while corresponding to nearly identical systems. For our work, the data thus have to be pre-processed to become invariant with respect to translations and rotations. Furthermore, the ML problem also has to be atom-index-invariant, and the sorting method also must be reversible to allow back-mapping of the features indicated by the ML to the relevant atom (or atom pairs).

In the present work, we considered atomic distances and velocities as possible features. Since the atomic velocities did not provide a significant contribution in our results, the forthcoming analysis has been based on atomic distances only.

The translation and rotation-independent requisites might be met with an atom–atom distance matrix. The atom-index-invariant approach requires, on the other hand, a more elaborate representation. First, a reference atom, which can differ in different frames if the atoms are indistinguishable (*i.e.*, the same element in an atomistic simulation), shall be selected. Thereafter, the rows in the atom–atom distance matrix are grouped per element and sorted within each element-group based on the distance from the reference atom. For each row in the matrix, the columns are grouped and sorted following an analogous procedure. The sorting is thus based on the distance from the atom indicated by the row. The column indices can therefore indicate a different atom for each row. A “′” denotes the secondary index.

**Figure 2:**
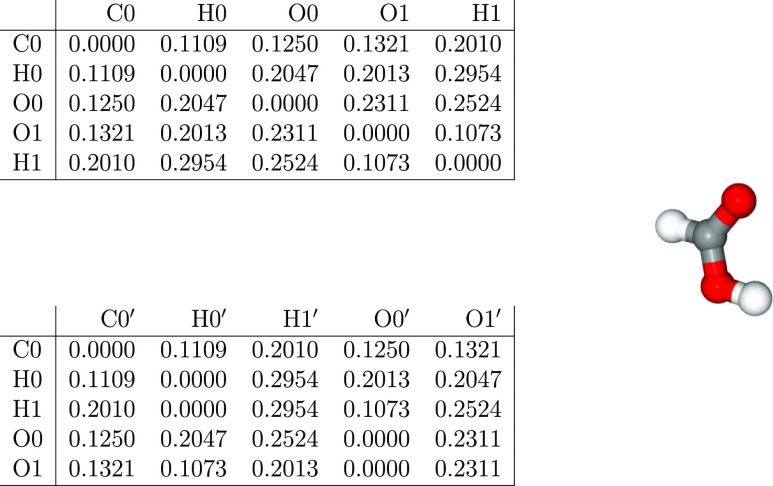
Distance matrix (top) for a structure of FA (right). The index-invariant distance matrix (bottom) corresponds to the first distance matrix. The prime on the column atom index indicates that it depends on the row atom index.

The resulting matrix reports the distance from a selected reference atom (rows) to its next neighbor (columns). A scheme of the algorithm to generate both the distance matrix and the index-invariant distance matrix is provided in the Supporting Information. In Figure 2, the two matrix representations are provided, as an example, for an isolated FA molecule, where we used the carbon atom (C0) as the trivial identifiable reference atom. The resulting internal coordinate representation allows an independent analysis of each entry and, thus, a suitable data structure for the ML task.

We would like to note here that a common translational- and rotational-invariant representation, the *Z*-matrix,^52^ also provides an appealing internal representation of molecular structures as it scales better with the number of atoms compared to the distance matrix. However, the values of its variables (*i.e.*, distances, angles, and dihedrals) are dependent on each entry and on the atom sequence. In contrast, in our distance matrix representation, each entry is independent. Also, distances are unique, with a lower bound (0) and an upper bound (system size). These three characteristics allow for a suitable split of sample space by the DTs. Furthermore, our representation is index-invariant.

We here report the results obtained by the index-invariant distance matrix, which is the most general approach, even if more computationally demanding. It is worth noting that the index-variant distance matrix can be advantageous for its simplicity and symmetry in certain applications, for example, in the presence of atoms that do not swap order during a transition. The results for the index-variant distance matrix are presented in the Supporting Information.

Computationally, a DecisionTree Classifier from scikit-learn^53^ has been fed with the index-invariant matrix, flattened to a feature vector, using the “entropy” splitting criterion and a maximum depth of three.

### 2.7 Data Matrix Notation and DT Visualization

The atom labeling system we use identifies each atom with a character and a digit. The character corresponds to the atom type, while the digit corresponds to the position of the sorted distance list per element with respect to a reference atom, with the indexing starting at 0. The digit of the first entry in the atom–atom distance label refers to the sorted distance list with respect to the reference atom (C0). The digit of the second entry refers to the sorted distance list with respect to the first atom of the atom–atom pair. To highlight it, a prime (′) has been added to the second index. As two examples, (a) O2–H5′ corresponds to the distance from the third closest oxygen (O2) to the C atom to the hydrogen atom, which is 6th closest to O2. (b) H0–O0′ is the distance from the H closest to the C (H0) to the oxygen closest to H0.

A symmetric distance matrix can be back-mapped to *xyz* coordinates (up to a translation and rotation) as described by Young and Householder^54^ (and further detailed in the Supporting Information). The index-invariant distance matrix can be unsorted into the symmetric matrix up to an atom index difference. The approach permits the addition of dummy atoms according to the splits given by the DT, allowing a direct visualization of the analysis output (*e.g.*, *via* VMD^55^). For a convenient visualization, only the dummy atoms corresponding to the nodes along each decision path in the tree might be selected. A main decision path is chosen such that a leaf node would have the highest number of pertinent reactive paths weighted by the percentage of pertinent reactive paths: *n*_r_·*n*_r_/(*n*_r_ + *n*_u_), where *n*_r_ is the number of reactive paths in that node and *n*_u_ the number of unreactive paths.

### 2.8 Random Forest Decision Error Estimate

The prediction error is simulation time-dependent and the true answer is unknown. Furthermore, due to time evolution, the distribution is not Gaussian and the noise is heteroscedastic with respect to the true value. Our implementation of the DT algorithm is not designed to make statistical predictions; instead, it focuses on identifying the most important features (regularization). To provide an estimate of the method’s reliability in the feature selection, an error-estimate procedure has been thus developed.

With highly correlated data, significantly different trees can be originated depending upon the first split from minor variations of the input. It is a constitutive limitation of the approach. The relative importance of the first split can be asserted by using random forests^56^ with a unit depth. The random forest reports the importance of all features by sampling several DTs, each one generated from a subset of features. By limiting the depth of each tree to 1, the feature importance of such random forests becomes equal to the importance of the first split only. It shall be noted that the feature that has the highest importance in the random forest plot does not necessarily represent the main split for all possible DTs.

A sequence of forests of DTs has been generated with respect to the time sequence during which sampling output has been generated. Source data have been split into 10 sub-blocks and randomized within each. A random forest has then been computed for each of these subsets, generating a sort of time-dependent profile for the main splits, which allows the computation of a variance σ for each of the main features. The average value for each feature can then be computed by considering the whole data set. By assuming a Gaussian distribution for each feature and by using the previously obtained variance and mean value for each feature, the relative probability of a feature importance is estimated. By comparing the probability distribution for each feature, the most relevant can be identified even if the data are highly correlated.

Computationally, a RandomForest Classifier from scikit-learn^53^ has been fed with the index-invariant matrix, flattened to a feature vector, using the “entropy” splitting criterion and a maximum depth of one.

## 3 Results and Discussion

**Figure 3:**
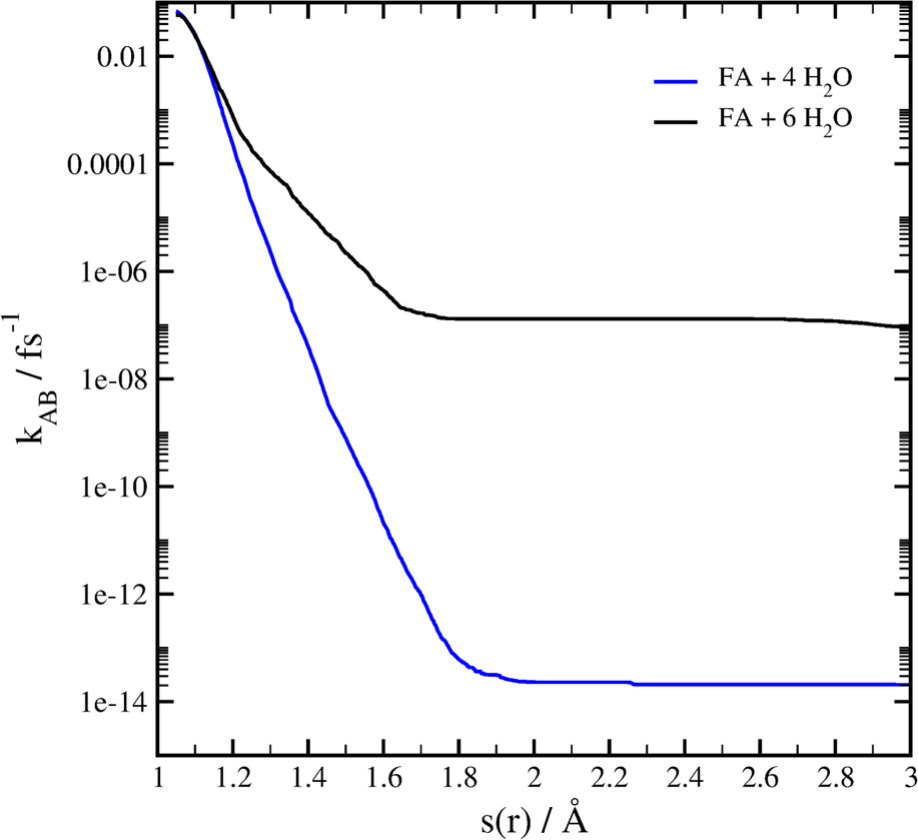
Effective rate constant *k*_AB_ computed using RETIS for FA clustered with four or six water molecules to reach the deprotonated state. Results are obtained from an average of several RETIS simulations from different initial conditions weighted by the respective number of RETIS cycles.

The rate of proton transfer from FA to the water molecules has been computed *via* RETIS simulation and *ab initio* MD simulations. Figure 3 reports the rate of reaction for two systems, where four and six water molecules surrounded FA. State A (protonated state) is defined as configurations with *s*(*r*) < 1.05 Å (four waters) or with *s*(*r*) < 1.07 Å (six waters). State B includes configurations with *s*(*r*) > 3.0 Å for both systems.

The rate of proton transfer for the four-water-molecule case is ∼2.10 × 10^–14^ and ∼1.01 × 10^–7^ fs^–1^ for six water molecules around FA (10^7^ times difference).

We here investigate the mechanism of reactions *via* DTs to identify the feature(s) that better correlate for each case with pathways that lead to proton transfer. The analysis might provide qualitative and quantitative descriptions of the different system features responsible for the significant difference in the reported rates.

### 3.1 FA with Four Water Molecules

**Figure 4:**
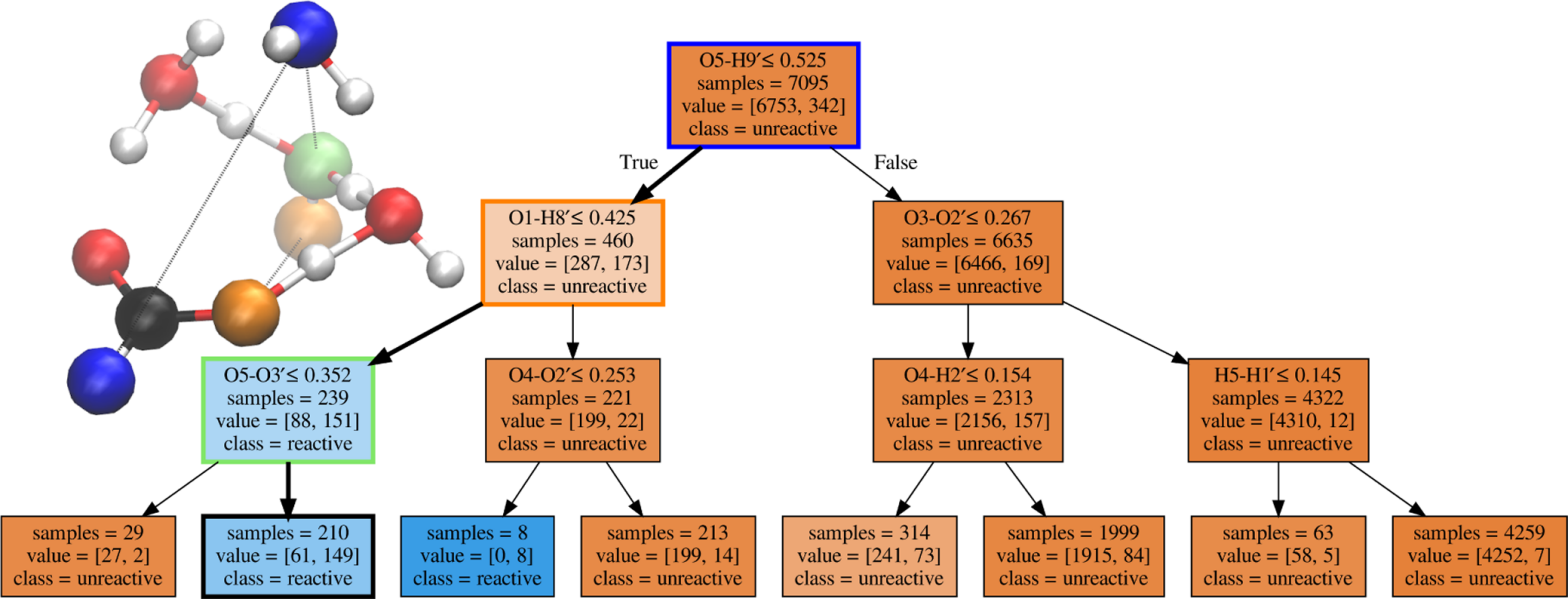
DT for the system with four water molecules around the FA molecule based on the index-invariant distance matrix. Each text box represents one node and reports (1) the inequality which splits the data going out of the node, (2) the number of samples entering the node, (3) the number of (unreactive, reactive) samples entering the node, and (4) the majority class of the node (*i.e.*, whether most of the data entering represent unreactive or reactive trajectories). At each split, the “True” branch is on the left and the “False” is on the right. The color indicates the ratio between unreactive (brown) and reactive (blue) samples included in a node. Wider arrows have been used to link the sequence of data splittings determined to be the most important in the analysis. In the top left corner, a 3D representation of the system is provided. The atoms highlighted in blue, yellow, and green correspond to the atoms involved in the first, second, and third split of the most important decision branch, respectively. In red, white, and black are the oxygen, hydrogen, and carbon atoms if not already highlighted as the most important decision branch.

The DT generated for the system with four water molecules clustered around FA is reported in Figure 4. To simplify the visualization of the main splits that lead to the highest reactive trajectories of the DT, in Figure 4, a Cartesian/*xyz* representation has been included. The atoms in blue, yellow, and green are involved in the first, second, and third splits, respectively.

The deprotonation reaction of FA appears to primarily require that the distance between O5 and H9′ be smaller than 5.25 Å. The split implies that the distance between the oxygen furthest from the FA carbon (O5) and the furthest hydrogen from O5 should be within a given threshold. As H9′ is the hydrogen of FA, it also implies that a certain orientation of the molecule, with respect to the water cluster, is also required. Under these conditions, the probability for the path to be reactive is 38%.

The next split along the branch with the highest probability to be reactive is the distance between O1 and H8′ being smaller than 4.25 Å. The distance between one of the FA oxygens and one of the furthest hydrogen atoms should be sufficiently small. This implies that the oxygen of FA should be located around the center of the cluster and that a sort of ordered disposition of the water molecules in the cluster is required. When this condition is satisfied, the probability for a path to be reactive reaches 63%.

Continuing along the branch with the highest reactive probability, the distance between O5 and O3′ being bigger than 3.52 Å represents the last split here considered. This corresponds to the relative position of two water molecules being two hydrogen bonds apart. We interpret the requisite as the suitable distance to establish hydrogen bonding between the atoms.

**Figure 5:**
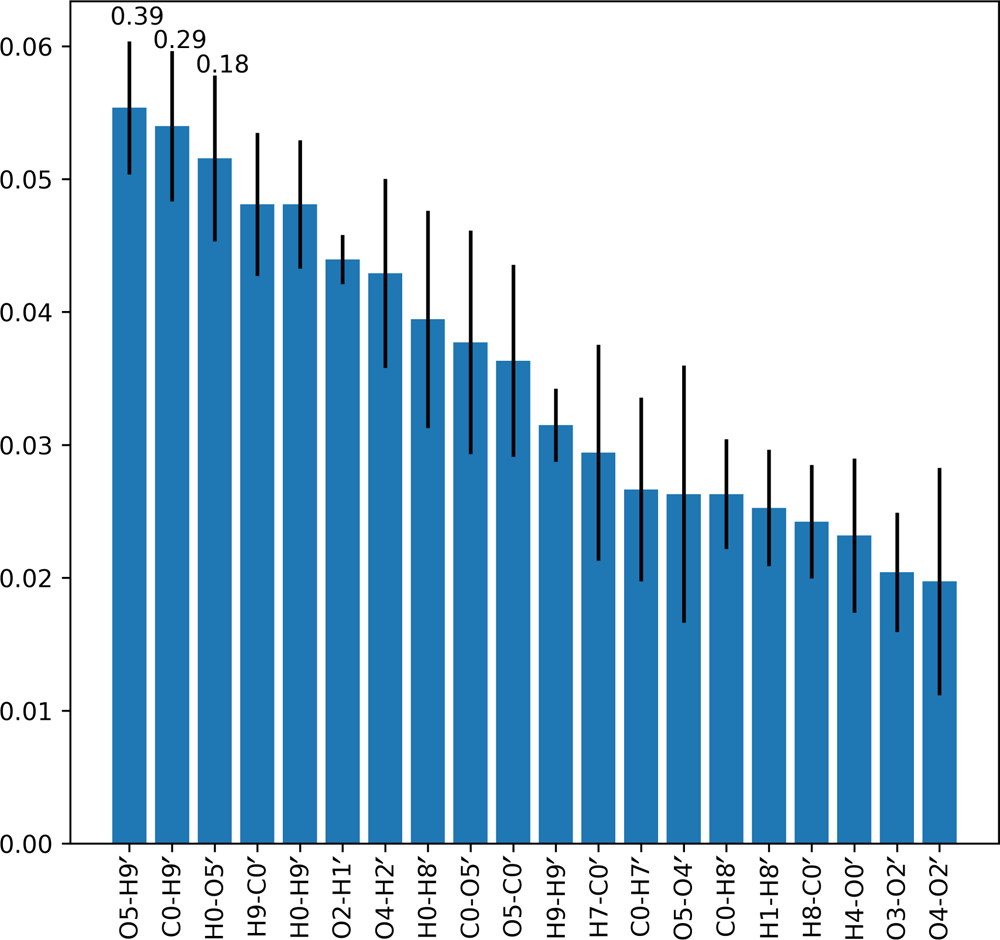
Importance approximations of the possible first split inequalities generated from a random forest with a depth of one for the four-water system. The bars represent the feature importance of a random forest, with the error bar calculated with a block-error average based on the generated trajectories. The probability that a split is truly the most important split is shown above the bars for the three most probable first splits.

When all three of these requirements are met, the probability of a path being reactive is 71%. By comparing the number of reactive paths versus the number of unreactive paths in the final splits of the DT, it can be concluded that the indicated reactive path is clearly predominant. A similar conclusion can be reached by observing the first splits reported in Figure 5. The figure that reports the results obtained from a random forest of DTs of depth 1 indicates that the relative probability for the first split to be the most important feature is 39%. The subsequent distances reported by the random forest analysis have a constantly decaying relevance. The first five main splits reported by Figure 5 are correlated and taken together indicate that the water cluster has to be sufficiently compact and FA has to be oriented such that its oxygen molecules are in close contact with the surrounding water molecules.

### 3.2 FA with Six Water Molecules

**Figure 6:**
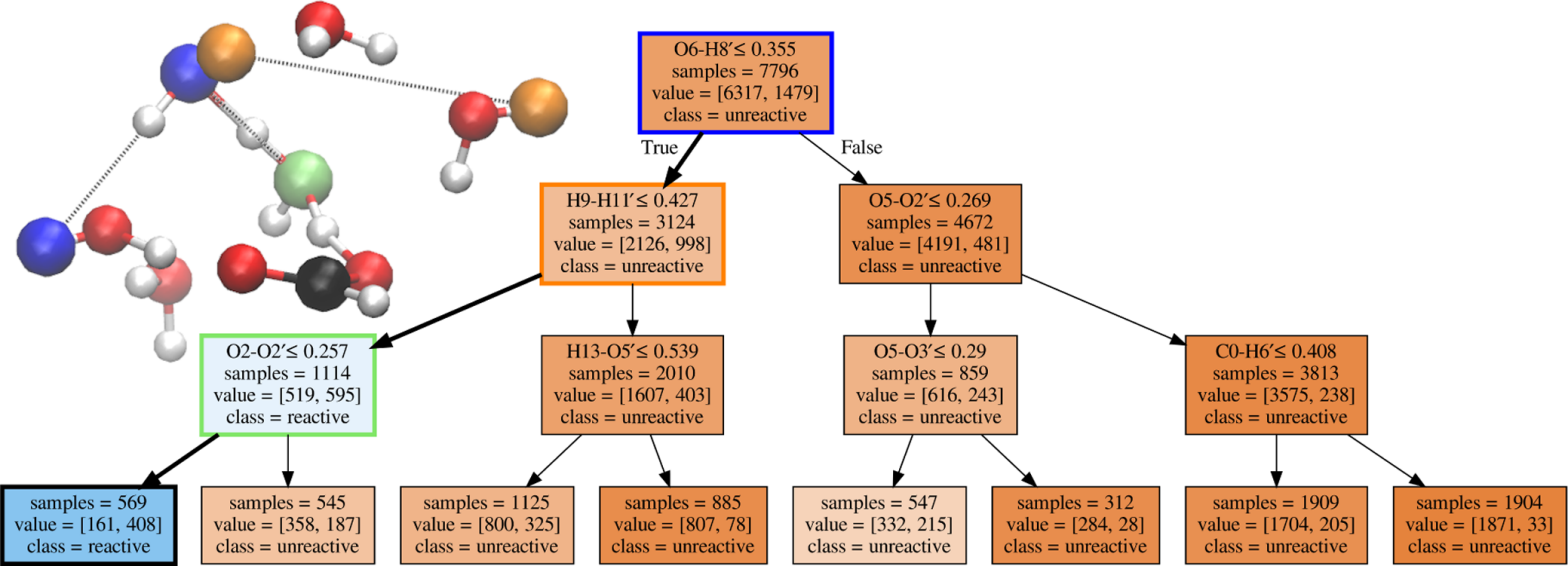
DT for the system with six water molecules around the FA molecule. For details, see the caption for Figure 4.

For the clusters with six water molecules around FA, in Figure 6, we report the generated DT. As we did with the four-water-molecule case, a visualization of the main splits of the DT that led to the highest reactive trajectories is also included in Figure 6. The atoms in blue, yellow, and green are involved in the first, second, and third splits, respectively.

The deprotonation reaction of FA in the six-water-molecule cluster requires the distance between O6 and H8′ to be smaller than 3.55 Å. This split involves two water molecules in the proximity of FA that need to be within a certain distance. By inspecting the frame reported in Figure 6, the requirement seems to indicate a certain orientation of one of the water molecules associated with another water molecule in proximity to the FA oxygen. Under such conditions, the probability for the path to be reactive is 32%.

The next split, along the branch with the highest probability to be reactive, is the distance between H9 and H11′ being smaller than 4.27 Å. The distance between these two atoms can also be interpreted as a combination of molecular orientation of the water molecules in the surroundings of FA and the water cluster size. The probability of a reactive path reaches 53% when both these conditions occur.

Still along the branch with the highest probability to be reactive, the distance between O2 and O2′ being bigger than 2.57 Å represents the last split here considered. This indicates that the closest water oxygen to FA (O2) should be close enough to its second closest oxygen atom to promote the formation of a hydrogen bond network. When all three requirements are met, the probability for a path to be reactive is 72%.

**Figure 7:**
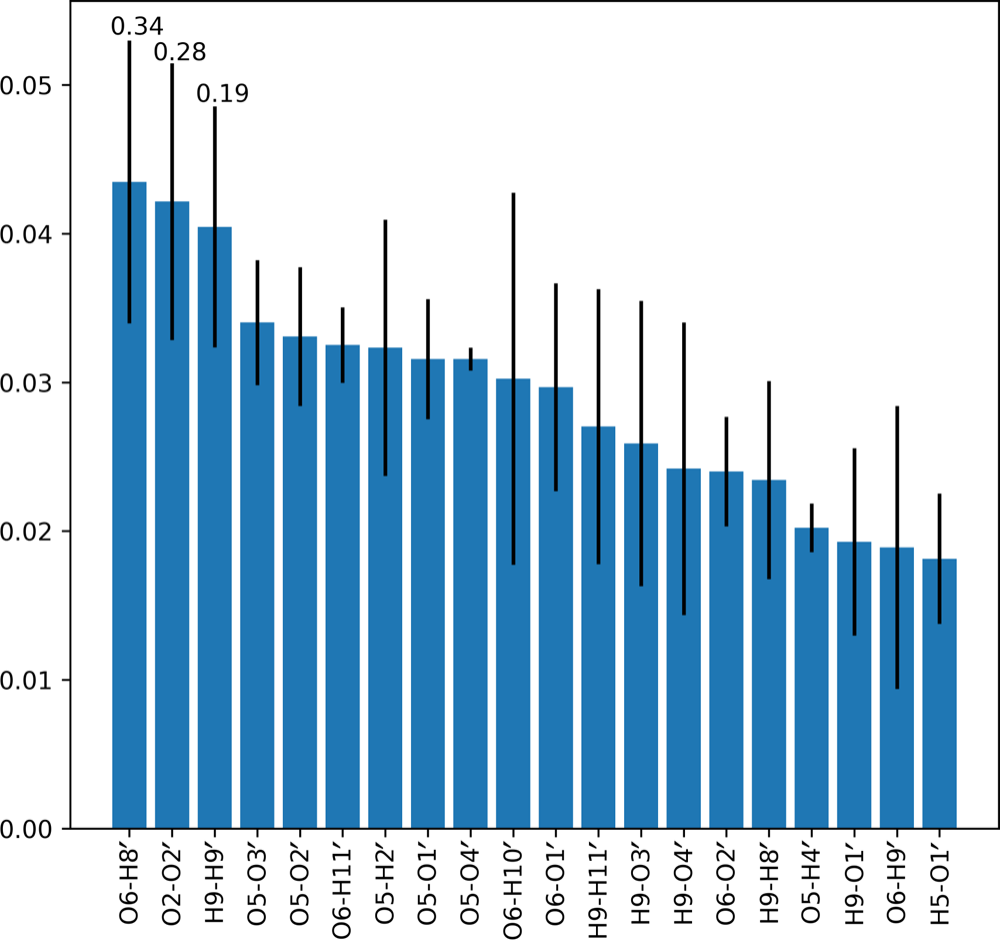
Importance approximations of the first split question from a random forest with a depth of one for the six-water system. For details, see the caption to Figure 5.

By comparing the number of reactive paths versus the number of unreactive paths in the final splits of the DT, it can be concluded that the indicated reactive path is clearly favorable, but that other significant paths also exist. The conclusion is also supported by the random forest of DTs with a single split. Before proposing an interpretation, it is worth the reminder here that the random forest reports unconditional entries, while the DT splits depend on the first split. Figure 7 indicates the probability that the first split is the most important feature is 34%, but the second split has a comparable relevance: O2–O2′ (28%). It confirms that while a predominant pathway for the reaction has been sampled, different main pathways can co-exist.

As reported in Figure 3, the number of water molecules in the cluster has a significant effect on the rate of the proton transfer reaction. From the comparison of the previously discussed Figures 4 and 6, we note that the distance between a FA oxygen and one of the furthest water hydrogens being below some distance is the predominant characteristic for a trajectory to be reactive. In other words, both clusters have to be sufficiently compact in order to promote the reaction. In the four-water-molecule case, the orientation of FA with respect to the water cluster is the most important feature, while for the six-water-molecule case, the water structure around FA appears to be the predominant feature.

A second main difference between the four- and six-water cases is the possible pathways for the reaction to occur. The smaller system has only one predominant reactive path, while for the six-water-molecule cluster, multiple paths appear to co-exist, contributing to the final reaction rate. Physically, if the system is sufficiently large, different configurations can lead to the proton transfer reaction, consistent with the observation that the overall rate is much higher.

### 3.3 Computational Cost, Scaling, Method Transferability, and Limitations

The computational cost of our method is negligible in comparison to the cost of generating pathways *via* MD. It is worth stressing here that our approach does not aim to replace the generation of trajectories but to improve the description of their characteristics.

The time required to train the DT scales as a function of the number of frames and features. The required time scales as *O*(*N* log *N*), where *N* is the number of frames, and linearly with the number of features. For the six-water-molecule system, with 529 features and 11,418 frames, the training time required was 1.7 s on a laptop (Dell XPS-15 with an Intel i7-8750H, 6 cores, and 12 threads). The number of features in the proposed representation scales as *O*(*M*^2^), with *M* being the number of atoms, which could be an issue in both memory and computational time for relatively large systems. However, the training of the DT can be efficiently parallelized over the number of features, with only one communication step per split of the DT.

The trained DTs are generally not transferable to other systems for predictions. However, the training of DTs is efficient and the training input of the DT is a feature vector that can be generated directly for any atomistic system as long as the positions and elements of the atoms in a frame and the classification of the trajectory are known. The feature vector can also be extended with user-defined features. Therefore, our described data representation and training/analysis approach can be directly applied to other atomistic simulations.

One main limitation of the presented analysis method (as with any ML/data-driven method) is the effect of “garbage in, garbage out”. We aim to identify the most relevant features for a transition in a simulation. When the configuration data (the proposed feature space) do not properly correlate with the system dynamics (in the presence of underlying potential energy bias as in meta-dynamics simulation^57^) or when frames are more correlated to a source sub-set (*e.g.*, forward-flux-sampling^58^), the DTs still identify the most important feature for the classification, although the feature may be different from unbiased simulation.

## 4 Conclusions

A data-driven method to systematically compute reaction pathways has been presented. The conventional Cartesian/*xyz* data representation employed in molecular simulations is converted into an index-invariant distance matrix representation, which is also translation- and rotation-invariant. Thereafter, an approach which limits the correlation between elements in the source data (MD trajectories) has been proposed in conjunction with a rare event simulation framework. The data have then been fed to a supervised classifier method, the DT.

To simplify the interpretation of the classifier, a back mapping procedure from the index-invariant matrix has been adopted to emphasize the atoms involved, with each split identified by the DT. Generation of a random forest of DTs, in combination with block averaging, provided an error range for the first split of the DT.

We thus presented a data-driven approach to gain insight into a chemical reaction. The method has been designed such that it is readily applicable to other simulation strategies and types of transitions. The strength of the present approach is that it allows the use of complex collective variables which may be discontinuous and the estimation of the probability of their occurrence in a transition path. The descriptors to elucidate transition mechanisms might be directly implemented in a prediction method.^37^

The method adopted an index-invariant distance matrix providing a data-driven insight into the reaction pathways. The data-driven identification aims to identify interpretable pathways in a system composed of indistinguishable molecules. Applications to more inhomogeneous systems would be straightforward, especially if only a portion of the system atoms are of interest. The latter case would combine human intuition with a data-driven approach, which would, possibly, provide a better insight into the reaction if, and only if, the introduced bias is correct. Our method can be further expanded by considering a higher number of descriptors alongside the distance matrix. Velocities, angles between molecules, coarse-graining procedures, or a mix of user-defined functions^59^ could be fed into the DT and subsequent analysis.

To demonstrate the capabilities of the developed method, a mechanistic description of the proton transfer reaction in small aqueous clusters of FA has been provided. The reaction has been simulated *via* rare event simulation (replica exchange transition interface sampling^31^) and its rate quantified for two water clusters, one composed of four and one of six water molecules surrounding an FA molecule.

The reaction rate we computed is strongly influenced by the number of water molecules present. Mechanistically, the four- and six-water proton transfer reaction requires the water cluster to be sufficiently compact. The four-water-molecule system requires a certain orientation of the FA molecules and of the water molecules in its proximity. For the six-water-molecule case, a certain orientation of the outer water molecules appears to be more significant in describing the reaction path. Furthermore, the four-water cluster system indicated only one predominant pathway for the reaction to occur, while in the six-water-molecule cluster, several pathways have been identified, contributing to the higher reaction rate in this system.

## Supporting Information Available

It contains the algorithm to generate the atom index-invariant data representation, the theoretical background for mapping the symmetric distance matrix back to *XYZ* coordinates, and the analysis based on the index-variant distance matrix for FA with four and six water molecules (PDF)
