# Supplementary material for: Chemistrees: Data-Driven Identification of Reaction Pathways via Machine Learning
Source: J Chem Theory Comput. 2021 Sep 24;17(10):6193–202. doi: 10.1021/acs.jctc.1c00458 (PMC8515787; doi:10.1021/acs.jctc.1c00458)
Supplement: Supplementary file 1 — ct1c00458_si_001.pdf [file ct1c00458_si_001.pdf]

SI

Chemistrees: data driven identification of reaction pathways via machine learning

Sander Roet,*,† Christopher D. Daub,‡ and Enrico Riccardi¶

†*Department of Chemistry, Norwegian University of Science and Technology, Trondheim, Norway*

‡*Department of Chemistry, University of Helsinki, P.O. Box 55, FI-00014, Helsinki, Finland*

¶*Department of Informatics, UiO, Gaustadalléen 23B, 0373 Oslo, Norway*

E-mail: sander.roet@ntnu.no

Making the data atom-index invariant

In a condensed system, the atoms might swap order during a transition. An index invariant data representation is, therefore, not clearly advantageous since it requires extra processing and it cannot use a symmetric representation. Yet, as it constitutes the most general case, it has been considered in addition to the index-variant representation we use in the main text.

As shown in Figure 1, by choosing a "anchor" atom, which may be different for each frame, the data representation can become invariant with respect to translation, rotation, and changes in the atomic indices. For the FA in the water system the carbon atom is the trivial identifiable anchor. The rest of the atoms are sorted based on the atom type and the distance

from the anchor, as illustrated in Figure 2. The resulting data representation is atom-index invariant. Due to the statistical fluctuations in the atom positions, this procedure requires more data to achieve convergence of a ML algorithm, and imposes further requirements on the interpretation of the resulting random tree.

In our simulation, atoms of different indices do not swap places during transitions (They do, eventually, in the stable states). Therefore, we also reproduced our analysis with the relatively simple distance matrix.

Figure 2 reports a simplified algorithm to generate the distance matrix, while Figure 4 presents the algorithm to generate the index invariant distance matrix.

	C0	H0	O0	O1	H1
C0	0.0000	0.1109	0.1250	0.1321	0.2010
H0	0.1109	0.0000	0.2047	0.2013	0.2954
O0	0.1250	0.2047	0.0000	0.2311	0.2524
O1	0.1321	0.2013	0.2311	0.0000	0.1073
H1	0.2010	0.2954	0.2524	0.1073	0.0000

	C0	H0	O0	O1	H1
C0	0.0000	0.2010	0.1250	0.1321	0.1109
H0	0.2010	0.0000	0.2524	0.1073	0.2954
O0	0.1250	0.2524	0.0000	0.2311	0.2047
O1	0.1321	0.1073	0.2311	0.0000	0.2013
H1	0.1109	0.2954	0.2047	0.2013	0.0000

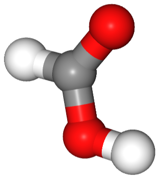

Figure 1: Two distance matrices (left) for an equivalent structure of formic acid (right). The difference between the two matrices is that H0 and H1 swapped places, or indices. This leads to a structure that has identical physics, but not an identical data representation. The distance matrix is therefore not a good representation to train our algorithms on during simulations where atom indices might change over time.

Back mapping the symmetric distance matrix to *xyz*

We adopted the procedure first suggested by Young and Householder [1]. If our distance matrix for a single frame is D_{ij}, we can construct the following mapping $M_{ij} = \frac{D_{1j}^2 + D_{i1}^2 - D_{ij}^2}{2}$, where

	C0	H0	H1	O0	O1
C0	0.0000	0.1109	0.2010	0.1250	0.1321
H0	0.1109	0.0000	0.2954	0.2013	0.2047
H1	0.2010	0.0000	0.2954	0.1073	0.2524
O0	0.1250	0.2047	0.2524	0.0000	0.2311
O1	0.1321	0.1073	0.2013	0.0000	0.2311

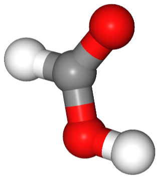

Figure 2: The index invariant distance matrix. It is created by first choosing an anchor point, in the present case, C0. Then the rows are grouped per element and sorted based on the distance from the anchor atom for each element. This representation is translationally, rotationally and atom-index invariant, making it a suitable general data representation to train ML algorithms on, including systems where atom indices might change.

Algorithm 2 Algorithm to group the symmetric distance matrix based on elements. Inputs: 'mat'=a symmetric distance matrix, 'Elements'=a list of all elements in the system. Outputs: 'out'=a symmetric distance matrix where all atoms of the same element are grouped together.

```
mat = distance_matrix
out = output_matrix
indices = List()
for element in Elements do                              ▷ group indices per element
    for atom in atoms do
        if atom.element=element then
            indices.append(atom.index)
        end if
    end for
end for
row_out, col_out= 0, 0
for row_i in indices do                                 ▷ Group elements together
    for col_i in indices do
        out[row_out][col_out] = mat[row_i][col_i]
        col_out + = 1
    end for
    row_out + = 1
end for
```

Algorithm 4 Algorithm to make the element grouped distance matrix atom-index invariant. Inputs: 'mat'=a symmetric distance matrix that has been grouped per element, 'Elements'=a list of all elements in the system, 'anchor_idx'=the row index which is the basis for the order of the rows in the output matrix. Outputs: 'out'=a sorted index-invariant distance matrix.

```
mat = grouped_distance_matrix                          ▷ Assume mat is grouped per element
out = output_matrix
elem_length = List(count(atoms of element e) for e ∈ Elements)
out_order = List()
anchor_idx = 0                                         ▷ Set anchor_atom to row 0
anchor_row = mat[anchor_idx]
i = 0
for j in elem_length do                                ▷ Figure out the output row-order.
    out_order.append(argsort(anchor_row[i:i+j]))
    i + = j
end for
row_out = 0
for row_idx in out_order do
    row = mat[row_idx]
    i = 0
    for j in elem_length do
        out[row_out][i:i+j] = sort(row[i:i+j])
        i + = j
    end for
    row_out + = 1
end for
```

D_{1j} is the j-th element of the first row of the distance matrix, and D_{i1} is the i-th element of the first column.

The eigenvalue decomposition on M: $M = USU^T$ allows the calculation of the matrix $X = U\sqrt{S}$. Only N of the eigenvalues (S) are non-zero for a system that can be embedded in N dimensional space, and distances are generated from a 3-dimensional space. Thus, the first 3 columns of X corresponds to the x, y, and z coordinates for each row or column in the original distance matrix, up to a translation or rotation.

Analysis based on the index variant distance-matrix

As the result obtained by the index invariant data matrix has been included in the main paper, only the results obtained from the index variant case have been included here.

Formic Acid with 4 water molecules

The frames identified by the selection windows have been transformed into the distance matrix representation (translational and rotational invariant) and fed to the machine learning algorithm to generate the forthcoming analysis. The only hyper-parameters are the location and size of the selection window.

Our analysis generated the decision tree reported in Figure 3A. To simplify the visualization of the main splits that lead to the highest reactive trajectories of the decision tree, in Figure 3B we report an *xyz* representation. The atoms in blue, yellow, and green are involved in the first, second, and third split, respectively.

The deprotonation reaction of FA appears to primarily require the distance between O0 and O3 to be smaller than 2.56 Å. In other words, the first water molecule that accepts the proton from FA has to be sufficiently close. When this condition occurs, the probability to obtain a reactive path rises from 5% to 12%.

From Figure 4 (bottom) the probability that this is the most important feature is 3%.

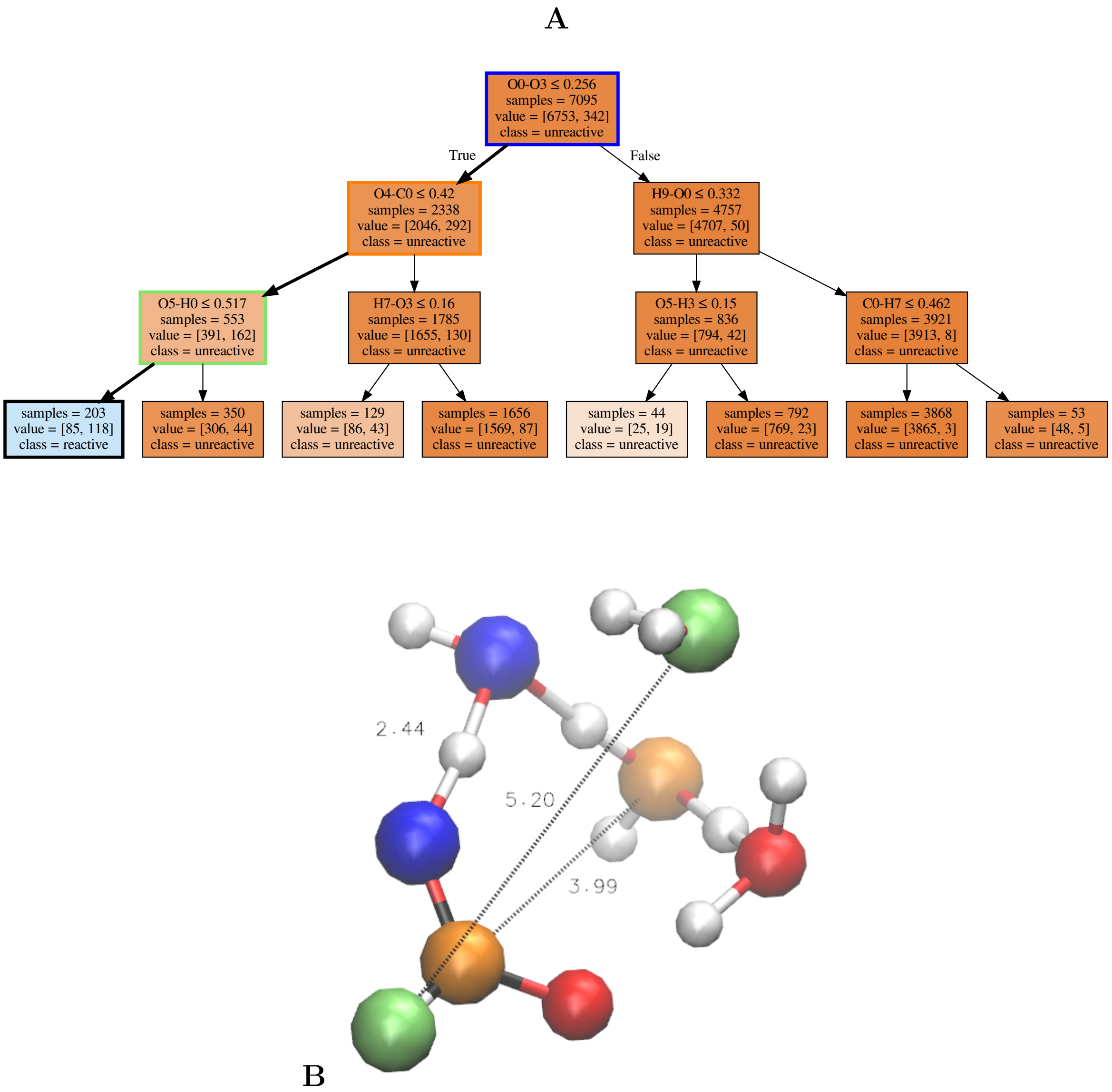

Figure 3: Decision tree for the system with 4 water molecules around the formic acid molecule based on the index-invariant distance matrix. Three splits divide the input, each square reports 1) the question to split the data, 2) the number of samples going into the node, 3) the number of [unreactive, reactive] samples going into the node and 4) the majority class of the node. At each split, the True branch is on the left, False on the right. The color indicates the ratio between unreactive (brown) and reactive (blue) samples included in a node. Wider arrows have been used to link the decision three split with the atoms involved. In panel B, a 3D representation of the system is provided. The atoms highlighted in blue, yellow, and green correspond to the atoms involved in the first, second, and third split of the decision three, respectively. In red are the oxygen and in white the hydrogen atoms not indicated by the decision tree reported in the panel B.

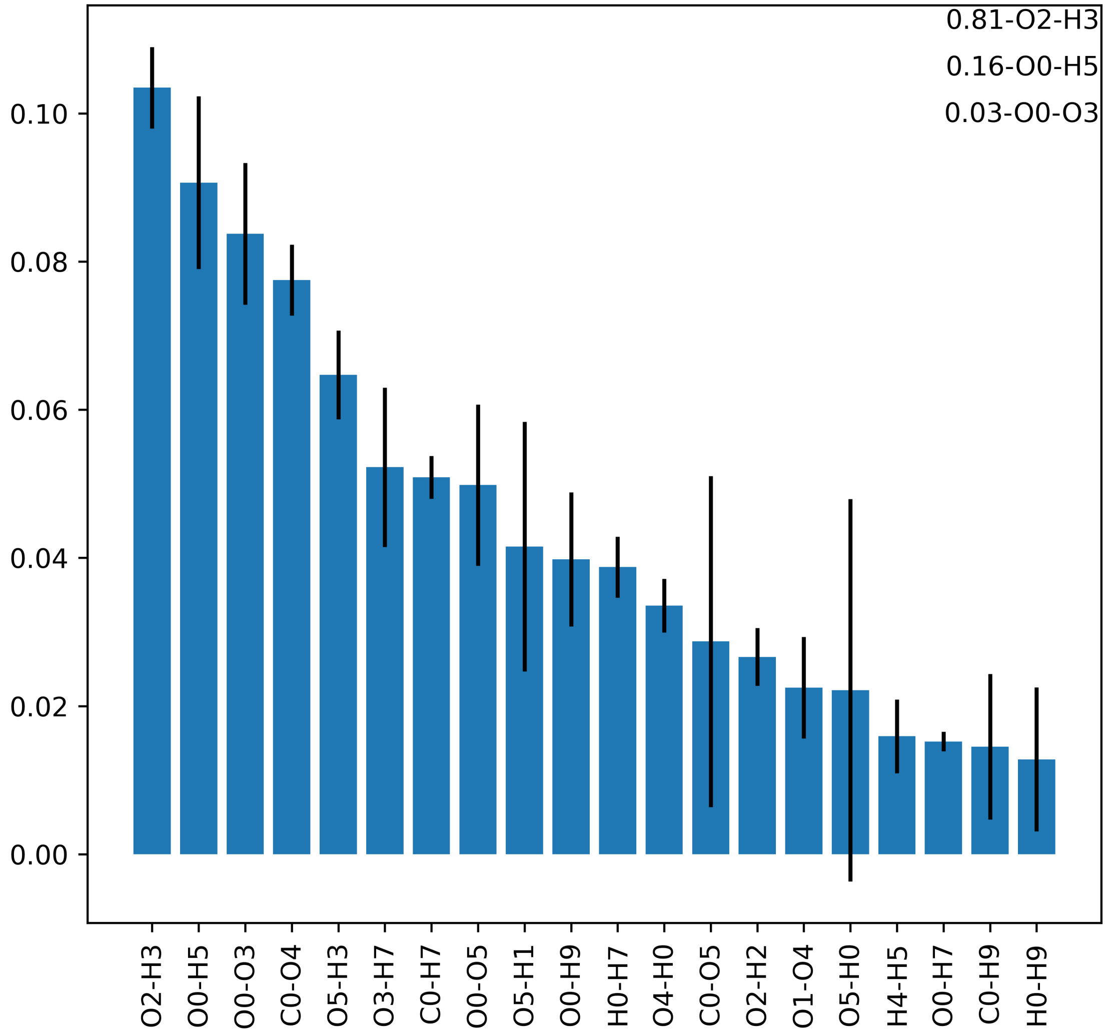

Figure 4: The importance approximations of the first split question from a random forest with depth of one for the four water case system. The bars represent the feature importance of a Random Forest, with the error bar calculated with a block-error average based on the generated trajectories.

Another feature has a probability of 16%. This is the distance between O0 and H5, the hydrogen that is connected to O3. Therefore, this condition is actually equivalent.

The next split, along the branch that leads to the highest reactive probability, is the requirement that the distance between C0 and O4 is smaller than 4.20 Å. Qualitatively, this means a water molecule has to be closer than two hydrogen bonds away from FA. If this second requirement is also satisfied, the probability of a reactive path is of 30%.

Still along the branch towards the highest reactive probability, the distance between O5 and H0 being smaller than 5.17 Å represents the last split here considered. This indicates that a second water molecule has to be within two hydrogen bonds away. When this additional requirement is satisfied, the probability of a reactive path reaches 58%.

Formic Acid with 6 water molecules

Figure 5 reports the analysis results obtained from the simulation of the proton transfer reaction for formic acid in a 6 water molecule cluster. In the figure, the decision tree and the *xyz* structure which highlights the most probable splits which determine a reactive path are included.

In this system the deprotonation reaction of FA appears to primarily require the distance between O7 and O2 to be smaller than 2.54 Å. The first water molecule that accepts the proton from FA has to be sufficiently close. When this condition occurs, the probability to obtain a reactive path raises from 19% to 44%.

From Figure 6 (bottom) the probability that this is the most important feature is 3%. One other equivalent feature has a probability of 16%, which involves the distance between O0 and H5, the hydrogen that is connected to O3.

The next split, still along the branch that led to the highest reactive stance, is the distance between O7 and O3 being smaller than 3.84 Å. If a second water molecule is sufficiently close to the FA oxygen, the probability of a reactive path is 60%.

Still along the branch towards the highest reactive stance, the distance between the

A

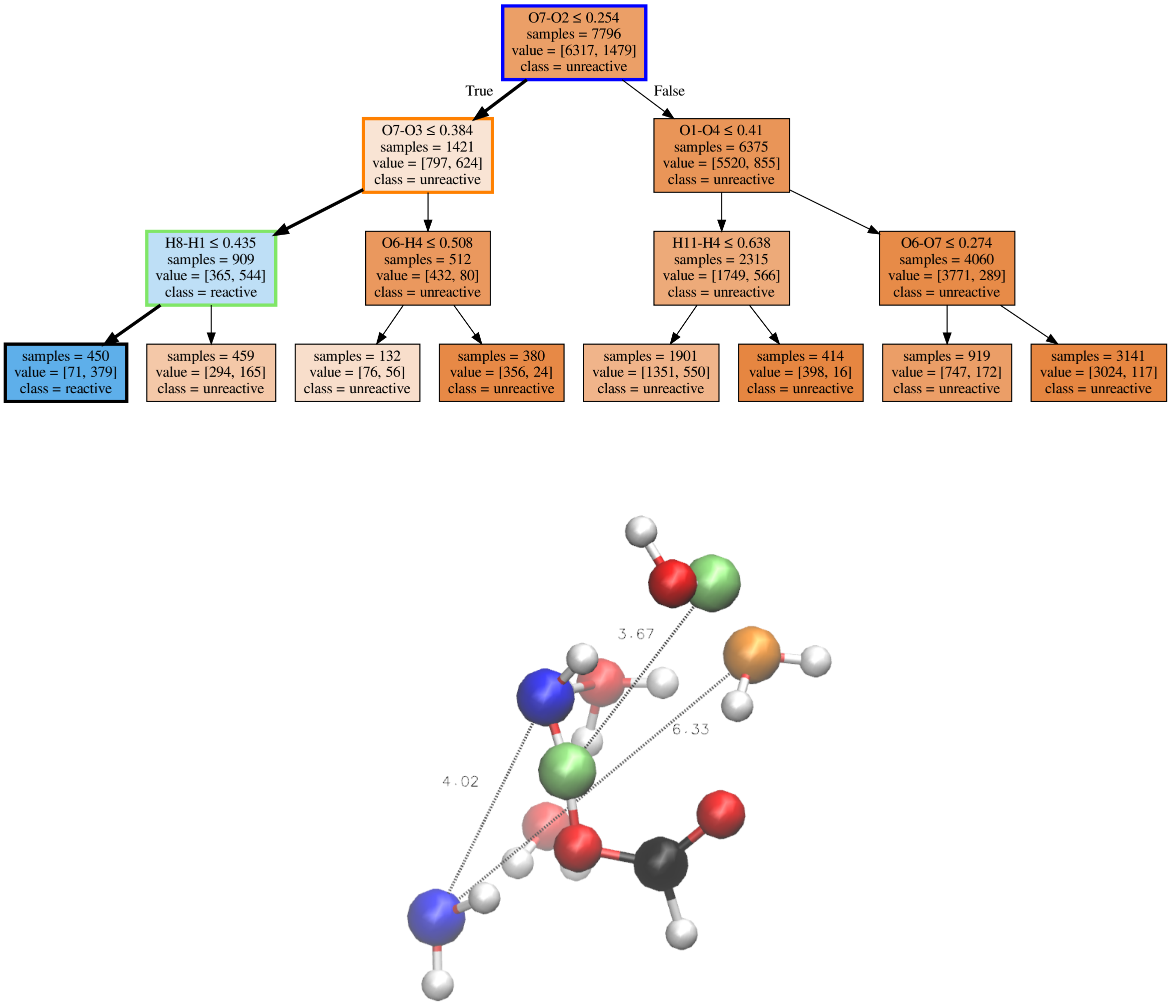

B

Figure 5: Decision tree for the system with 6 water molecules around the formic acid molecule. Three splits divide the input, each square reports 1) the question which splits the data, 2) the number of samples going into the node, 3) the number of [unreactive, reactive] samples going into the node and 4) the majority class of the node. At each split, the True branch is on the left, False on the right. The color indicates the ratio between unreactive (brown) and reactive (blue) samples included in a node. Wider arrows have been used to link the decision three split with the atoms involved. In panel B, a 3D representation of the system is provided. The atoms highlighted in blue, yellow, and green, correspond to the atoms involved in the first, second, and third split of the decision three, respectively. In red are the oxygen and in white the hydrogen atoms not indicated by the decision tree reported in the panel B.

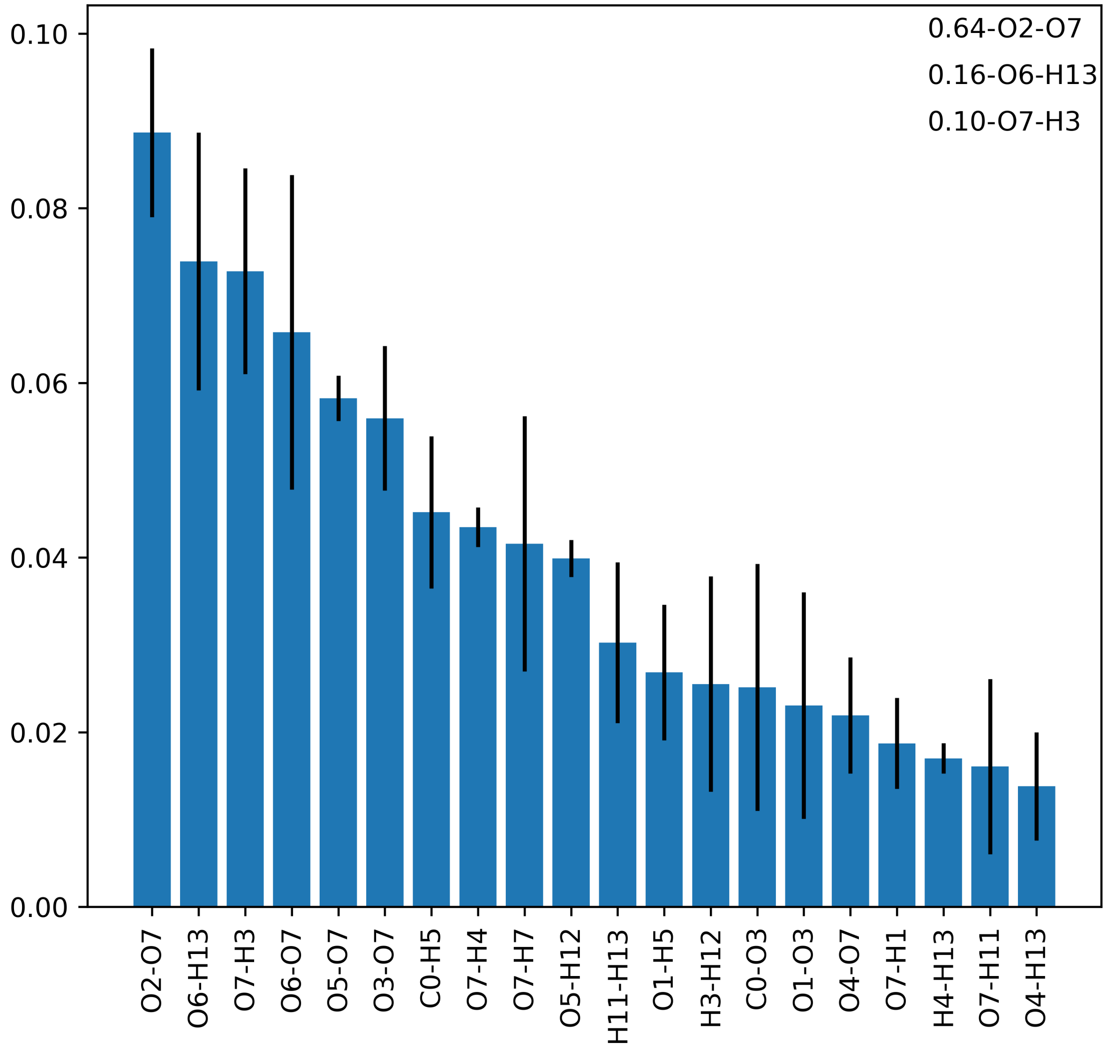

Figure 6: The importance approximations of the first split question from a random forest with depth of one for the four water systems. The bars represent the feature importance of a random forest, with the error bar calculated with a block-error average based on the generated trajectories.

hydrogen H8 and H1 being smaller than 4.35 Å represents the last split here considered. This feature can be interpreted as a structural requirement for the water complex to be reactive, and may also implicitly involve some requirement involving atomic orientations/angles. In such conditions, the probability for the path to be reactive is 84%.

The result reported by the decision trees generated by the index variant and index invariant representations are physically consistent but different in atom selection. That is, different atom pairs for the different representations are identified as most relevant. Yet, the water structure and formic acid orientation are equivalent. It should also be noted that the index variant representation results in a numerically more stable generation of random forests, as the importance values for the symmetric features are summed together. For the distribution of importance of the random forest reported in Figures 4 and 6, the first splits are thus identified with a higher probability.

References

(1) Young, G.; Householder, A. S. Discussion of a set of points in terms of their mutual distances. *Psychometrika* **1938**, *3*, 19–22.
